# Doppler Echocardiography-Guided Heart Rate Modulation Therapy Using Ivabradine in a Patient with Systolic Heart Failure

**DOI:** 10.3390/medicina58020164

**Published:** 2022-01-21

**Authors:** Teruhiko Imamura, Koichiro Kinugawa

**Affiliations:** Second Department of Internal Medicine, University of Toyama, 2630 Sugitani Toyama, Toyama 930-0194, Japan; kinugawa-tky@umin.ac.jp

**Keywords:** heart failure, reverse remodeling, hemodynamics

## Abstract

Heart rate reduction using ivabradine, a selective I_f_ channel blocker that purely decreases heart rate without affecting hemodynamics, improves clinical outcomes in patients with systolic heart failure. However, the ideal heart rate that should be a target remains unknown. Our team recently proposed a methodology using Doppler echocardiography to estimate ideal heart rate, at which E-wave and A-wave stand adjacent without overlap. However, the implication of Doppler echocardiography-guided heart rate modulation therapy using ivabradine remains uncertain. We had a 72-year-old man with systolic heart failure and sinus tachycardia who initiated ivabradine therapy. Ivabradine dose was adjusted between 5.0 mg/day and 10.0 mg/day and continued for 12 weeks to minimize the overlap between the two echocardiography waves, accompanying improvement in cardiac output, left ventricular ejection fraction, plasma B-type natriuretic peptide, and six-minute walk distance. Doppler echocardiography-guided heart rate regulation therapy using ivabradine may be a promising strategy to improve cardiac function and clinical outcomes in patients with systolic heart failure, although further studies are required to validate this hypothesis.

## 1. Introduction

Heart rate reduction using ivabradine, an I_f_ channel blocker that purely decreases heart rate without affecting hemodynamics, [1] improves mortality and morbidity in patients with systolic heart failure [2]. However, the ideal heart rate that should be a target of heart rate modulation remains unknown. Extremely lower heart rate rather decreases cardiac output and deteriorates hemodynamics, resulting in worse clinical outcomes [3].

Our team recently proposed the utilisation of Doppler echocardiography to estimate the ideal heart rate, at which trans-mitral inflow E-wave and A-wave stand adjacent without overlap and cardiac output is maximum without wasting cardiac potential energy [3]. However, the clinical implication of this methodology remains uncertain. 

We here present a patient with systolic heart failure, whose heart rate was successfully regulated by repeating Doppler echocardiography and adjusting ivabradine dose to minimize the waves’ overlap.

## 2. Case Report

### 2.1. Before Referral

A 72-year-old man with diabetes mellitus was admitted to the former institute complaining of dyspnea on exertion with New York Heart Association functional class IV. He had received coronary angiography 3 years ago and significant coronary artery stenosis was denied. He was diagnosed with pulmonary pneumonia and decompensated systolic heart failure, which were treated with antibiotics and intravenous diuretics. Carvedilol 2.5 mg/day and enalapril 1.25 mg/day were initiated before discharge.

### 2.2. After the Discharge

Following discharge, enalapril was converted to sacubitril/valsartan 100 mg/day. Dapagliflozin 10 mg/day and spironolactone 12.5 mg/day were initiated. Nevertheless, his heart failure symptoms, including dyspnea on exertion, remained. He was referred to our out-patient clinic to receive further treatments.

### 2.3. On Referral

On referral, he had dyspnea on exertion and was assigned to the New York Heart Association functional class III. The patient’s blood pressure was 108/63 mmHg and his pulse rate was 92 bpm. He had received carvedilol 5.0 mg/day, sacubitril/valsartan 100 mg/day, spironolactone 12.5 mg/day, dapagliflozin 10 mg/day, and furosemide 10 mg/day.

**Figure 1:**
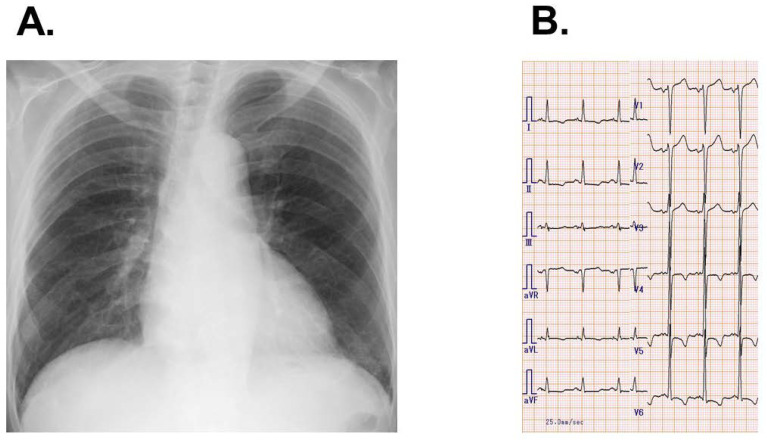
Chest X-ray (**A**) and electrocardiogram (**B**) on admission.

He had no obvious peripheral edema. A chest X-ray revealed mild cardiomegaly with cardiothoracic ratio of 0.54 and mild bilateral pulmonary congestion (Figure 1A). Electrocardiography showed normal sinus rhythm, high voltage, and negative T waves with strain pattern in V4-6 (Figure 1B).

**Figure 2:**
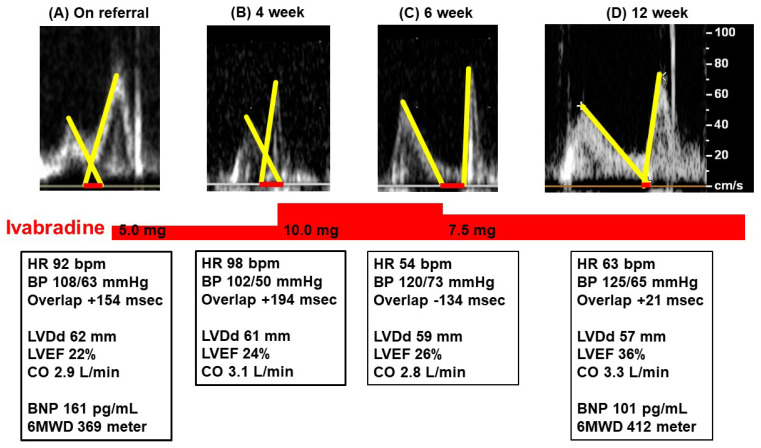
Trends in Doppler echocardiography trans-mitral inflow waves and clinical data on referral (**A**), 4 weeks later (**B**), 6 weeks later (**C**), and 12 weeks later (**D**). Ivabradine was initiated on referral and the dose of ivabradine was titrated to minimize the overlap length (red bars). HR, heart rate; BP, blood pressure; LVDd, left ventricular end-diastolic diameter; LVEF, left ventricular ejection fraction; CO, cardiac output; BNP, B-type natriuretic peptide; 6MWD, 6-min walk distance.

Transthoracic echocardiography showed a left ventricular end-diastolic diameter of 62 mm, left ventricular ejection fraction 22%, and a mild mitral regurgitation (Figure 2A). Intraventricular septum and posterior wall thickness were 8/9 mm. E/e’ ratio was 17.9. The overlap length between the two waves was +154 msec. Cardiac output was 2.9 L/min, which was estimated using AESCULON mini [4]. Plasma B-type natriuretic peptide was 161 pg/mL and six-minute walk distance was 369 m. He was suspected of dilated cardiomyopathy.

He complained of dizziness while on further up-titration of carvedilol and we initiated ivabradine 5.0 mg/day. By substituting the deceleration time 230 msec to the formula of ideal heart rate: 96 – 0.13 × (deceleration time [msec]) [3], a target heart rate was calculated as 66 bpm.

### 2.4. Follow-Up

Following the 4-week ivabradine therapy (Figure 2B), his heart rate remained 98 bpm. The overlap length was +213 msec. The dose of ivabradine was up titrated to 10.0 mg/day. At 6 weeks following referral (Figure 2C), his heart rate decreased to 54 bpm. E-wave and A-wave in the trans-mitral inflow stood apart. Cardiac output rather decreased to 2.8 L/min. He complained of recurrent dyspnea on exertion. The dose of ivabradine was decreased to 7.5 mg/day. 

Following the 12-week ivabradine therapy (Figure 2D), the patient’s heart rate was 63 bpm. The two trans-mitral inflow waves almost stood adjacent without overlap. Left ventricular ejection fraction increased to 36%. Plasma B-type natriuretic peptide decreased to 101 pg/mL. His heart failure symptoms improved accompanying New York Heart Association function class II and six-minute walk distance 412 m. He continued ivabradine therapy at 7.5 mg/day. 

## 3. Discussion

### 3.1. Ideal Heart Rate

In the SHIFT trial [2], the heart rate reduction therapy using ivabradine reduced cardiovascular death and heart failure readmission compared to the placebo in patients with systolic heart failure and sinus tachycardia (heart rate at rest ≥ 70 bpm). However, target heart rate during the heart rate regulation therapy remained uncertain [3]. 

Too fast heart rate would reduce cardiac output due to insufficient diastole phase, which is known as tachycardia-induced cardiomyopathy. An unnecessary cardiac contraction rather wastes potential energy. On the contrary, an extremely slow heart rate also decreases cardiac output due to insufficient cardiac contractile times, stimulating sympathetic nerve activity. Cardiac reverse remodeling would not be achieved in both too fast and too slow heart rate situations.

Our team recently proposed measuring the overlap between trans-mitral inflow E-wave and A-wave in the Doppler echocardiography to optimize heart rate [3]. At the ideal heart rate, both waves stood adjacent without overlap, achieving maximized cardiac output and future cardiac reverse remodeling.

In real-world clinical practice, the ideal heart rate, at which two waves had no overlap, was consistently associated with greater cardiac reverse remodeling and lower mortality and morbidity in various clinical situations, including systolic heart failure [5].

### 3.2. Aggressive Heart Rate Modulation

In a retrospective study, cardiac output was maximized when heart rate was modulated by ivabradine, and the 2-wave overlap reached around zero [6]. In another retrospective study, patients with systolic heart failure enjoyed greater cardiac reverse remodeling and lesser heart failure recurrence when their heart rate was within 10 bpm of the calculated ideal heart rate during ivabradine therapy, compared with those with sub-optimal heart rate control [7].

Given all together, we aggressively modulated the patient’s heart rate to minimize the overlap length by adjusting the dose of ivabradine. His heart rate decreased to 54 bpm by 10.0 mg/day of ivabradine. A heart rate between 50 and 60 bpm is, in general, considered to be appropriate. However, the two waves were apart in the Doppler echocardiography when his heart rate was 54 bpm, accompanying a rather decreased cardiac output and worsening heart failure symptoms. He eventually enjoyed incremental cardiac output, greater cardiac unloading and more facilitated cardiac reverse remodeling, as well as improved exercise capacity, when his heart rate was modulated around the ideal rate by the echo-guided ivabradine dose adjustment.

One limitation of this methodology is the requirement of repeated echocardiography. We proposed a formula to calculate the ideal heart rate by using baseline deceleration time [3]. In this patient, the calculated ideal heart rate on referral was 66 bpm, which was almost similar to the actual heart rate maintained at 12 weeks later. The calculation of ideal heart rate would be an alternative to repeated echocardiography if patients’ hemodynamics are relatively stable and their deceleration time is assumed to be constant.

## 4. Conclusions

We experienced a patient with systolic heart failure whose heart rate was successfully regulated by adjusting ivabradine dose under the repeated Doppler echocardiography guide. Doppler echocardiography-guided heart rate regulation using ivabradine might be a promising strategy to optimize heart rate and improve clinical outcomes, although further prospective randomized control studies are warranted to validate our strategy.

## Data Availability

Data are available from the corresponding author on appropriate requests.

## References

[1] Koruth J.S., Lala A., Pinney S., Reddy V.Y., Dukkipati S.R. (2017). The Clinical Use of Ivabradine. J. Am. Coll. Cardiol..

[2] Swedberg K., Komajda M., Bohm M., Borer J.S., Ford I., Dubost-Brama A., Lerebours G., Tavazzi L. (2010). Ivabradine and outcomes in chronic heart failure (SHIFT): A randomised placebo-controlled study. Lancet.

[3] Izumida T., Imamura T., Nakamura M., Fukuda N., Kinugawa K. (2020). How to consider target heart rate in patients with systolic heart failure. ESC Heart Fail..

[4] Nakayama A., Iwama K., Makise N., Domoto Y., Ishida J., Morita H., Komuro I. (2020). Use of a Non-invasive Cardiac Output Measurement in a Patient with Low-output Dilated Cardiomyopathy. Intern. Med..

[5] Izumida T., Imamura T., Ueno Y., Tanaka S., Kataoka N., Nakamura M., Kinugawa K. (2021). Impact of optimal heart rate on left ventricular reverse remodeling and functional improvement in patients with systolic heart failure. Heart Vessel..

[6] Hori M., Imamura T., Narang N., Kinugawa K. (2021). Implications of Doppler Echocardiography-guided Heart Rate Modulation Using Ivabradine. Intern. Med..

[7] Imamura T., Hori M., Narang N., Besser S., Kinugawa K. (2021). Prognostic implications of mitral valve inflow pattern overlap during ivabradine therapy. Int. Heart J..

